# MDA5-associated juvenile dermatomyositis and interstitial lung disease from rapidly progressive to silent: a report of three cases in South African children and a review of the literature

**DOI:** 10.1007/s10067-026-08070-6

**Published:** 2026-04-22

**Authors:** Maurane Lepage, Gabriella Pereira, Shehnaaz Akhalwaya, Taryn Gray, Marco Zampoli, Mignon McCulloch, Peter Nourse, Ashton Coetzee, Claire Procter, Khanyisile Hlongwa, Tanyia Pillay, Kate Webb

**Affiliations:** 1 Department of Paediatric Rheumatology, Red Cross War Memorial Children’s Hospital, University of Cape Town, Cape Town, South Africa; 2 Chest and Allergy Centre, Christian Barnard Memorial Hospital, Cape Town, South Africa; 3 Department of Paediatric Pulmonary, Red Cross War Memorial Children’s Hospital, University of Cape Town, Cape Town, South Africa; 4 Department of Paediatric Nephrology, Red Cross War Memorial Children’s Hospital, University of Cape Town, Cape Town, South Africa; 5 Paediatric Intensive Care Unit, Red Cross War Memorial Children’s Hospital, University of Cape Town, Cape Town, South Africa; 6 Nuclear Medicine, Red Cross War Memorial Children’s Hospital, University of Cape Town, Cape Town, South Africa; 7 Department of Radiology, Red Cross War Memorial Children’s Hospital, University of Cape Town, Rondebosch, Cape Town, South Africa; 8 Department of Paediatrics, Hospices Civils de Lyon, Lyon, France

**Keywords:** Africa, Anti-MDA5, Juvenile dermatomyositis, Rapidly progressive interstitial lung disease

## Abstract

**Background:**

Juvenile dermatomyositis (JDM) is a rare pediatric autoimmune disease. A distinct clinical phenotype is associated with anti-melanoma differentiation-associated gene 5 (anti-MDA5) autoantibodies, which are linked to features such as arthritis, ulcerative skin lesions, and a heightened risk of interstitial lung disease (ILD), including its rapidly progressive form (RP-ILD). Despite increased recognition of this phenotype in East Asian, European, and North American populations, significant gaps remain in understanding its pathogenesis, and no consensus has been reached regarding optimal treatment strategies. Moreover, data on anti-MDA5-associated JDM in African populations are nonexistent.

**Case presentation:**

We report the first three documented cases of anti-MDA5-positive JDM with ILD in African children. All patients exhibited characteristic extramuscular manifestations, and all had pulmonary involvement, which was rapidly progressive in two children, one of whom died. The clinical course, diagnostic findings, and treatment strategies are discussed in the context of existing literature.

**Methods:**

A review of the literature was performed to evaluate the prevalence, clinical presentation, and treatment approaches for RP-ILD in anti-MDA5-associated JDM across different populations.

**Conclusion:**

These cases highlight the wide heterogeneity of clinical phenotypes associated with anti-MDA5 autoantibodies in JDM. Given this variability, individualized monitoring and management strategies are essential to optimize outcomes.

Juvenile dermatomyositis (JDM) is a rare autoimmune disease in children, primarily characterized by proximal muscle weakness and distinctive skin manifestations. A subset of JDM patients, those with anti-melanoma differentiation-associated gene 5 (anti-MDA5) autoantibodies, presents with a unique clinical profile, which includes arthritis, ulcerative skin lesions, and an increased risk of developing interstitial lung disease (ILD) and, in some cases, rapidly progressive ILD (RP-ILD) [1].

The prevalence and clinical course of ILD in JDM patients with anti-MDA5 vary significantly depending on geographic and ethnic backgrounds. In East Asia, the prevalence of anti-MDA5 in JDM is notably high at 30%, and over 60% develop ILD, with more than one-third progressing to RP-ILD [2]. In contrast, a UK cohort found that 7.4% of JDM patients were anti-MDA5 positive, with 19% developing ILD and none progressing to RP-ILD [3]. Compared to other myositis-associated autoantibodies, anti-MDA 5 positive patients typically exhibited arthritis, weight loss, and dysphagia but milder muscle involvement and a higher likelihood of achieving disease inactivity within 2 years. Similarly, a North American cohort reported that 28% of JDM patients with anti-MDA5 developed ILD, with 6% progressing to RP-ILD [4].

Despite the growing understanding of anti-MDA5-associated JDM in various populations, there is a lack of data regarding African patients. Here, we present the first documented cases of three African children with anti-MDA5-associated JDM and ILD, highlighting the clinical features and disease course in this understudied population and provide a review of the literature on the RP ILD related to MDA5 to review the different treatments.

## Case presentation

### Case 1

**Fig. 1:**
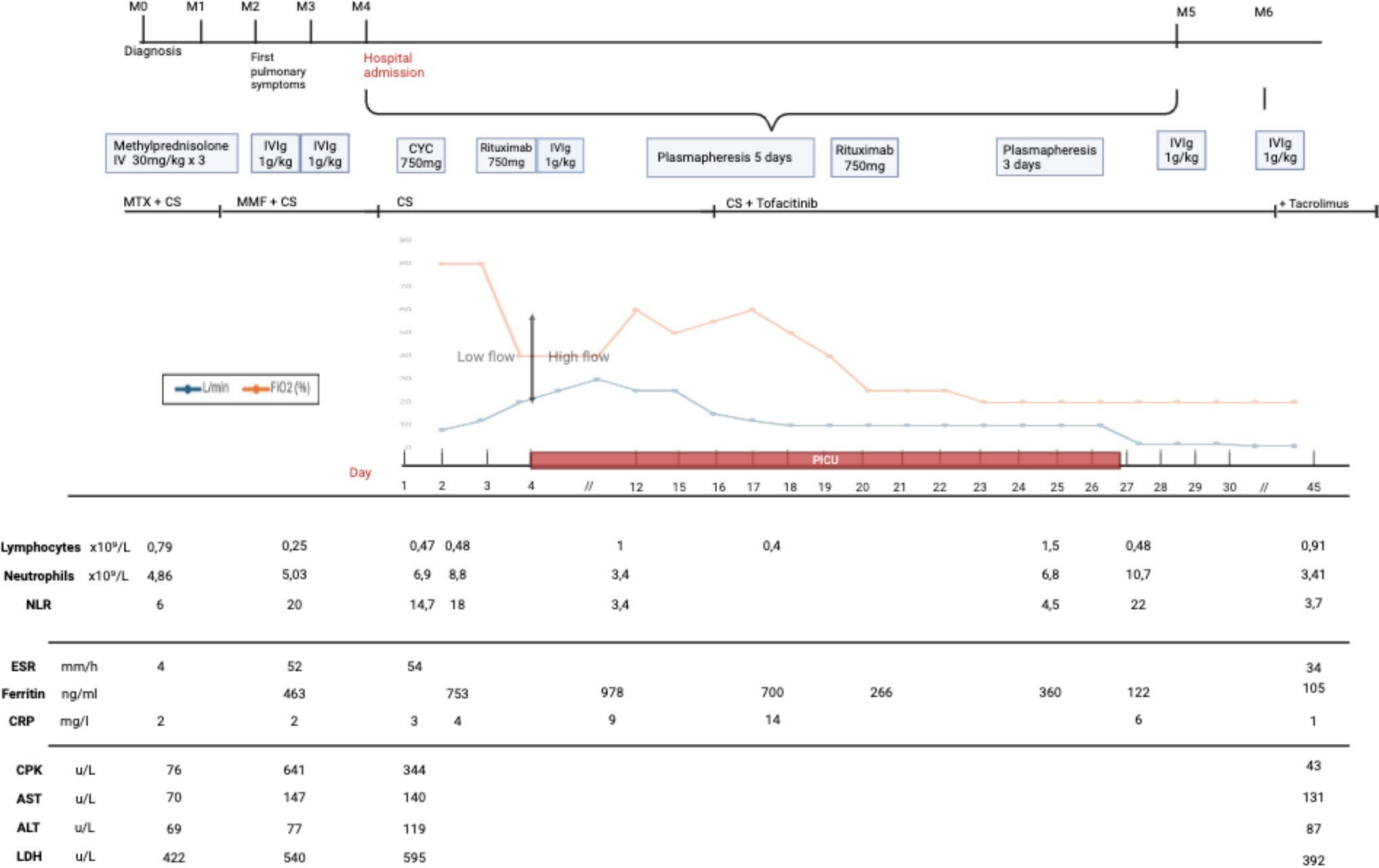
Clinical course of patient 1

An 11-year-old previously healthy boy, actively involved in water polo and saxophone playing, presented with a history of weakness and body pain. He was initially investigated for a malignancy due to a persistent lymphopenia. A bone marrow biopsy was performed which revealed a mildly hypocellular marrow with no significant myelodysplasia. Due to ongoing symptoms, he was referred to rheumatology (Fig. 1).

On physical examination, he was found to have arthritis, clinical muscle weakness (Childhood Myositis Assessment Scale [CMAS] score of 26/52), and characteristic dermatological features, including Gottron’s papules, heliotrope rash, and punched-out ulcers over the interphalangeal joints.

The diagnosis of juvenile dermatomyositis (JDM) was confirmed with myositis evident on muscle magnetic resonance imaging (MRI). Anti-MDA5 antibodies were detected by immunoblotting (dot blot assay for myositis-specific antibodies).

Initial treatment consisted of three pulses of high-dose, intravenous, methylprednisolone (30 mg/kg/pulse), followed by oral prednisone at 2 mg/kg/day and weekly subcutaneous methotrexate (15 mg/m^2^). One month later, methotrexate was discontinued due to transaminitis, and mycophenolate mofetil (MMF) was introduced as an alternative immunosuppressant. There was a modest improvement in muscle strength, but despite therapy, new ulcerative cutaneous lesions emerged. Intravenous immunoglobulin (IVIG) was added at a dose of 2 g/kg monthly. The patient subsequently demonstrated muscular improvement (CMAS 46/52), with no further skin ulcerations, allowing a gradual tapering of oral prednisone following PRINTO recommendations [5] to 1 mg/kg/day from week six and 0.5 mg/kg/day from week ten.

Three months post-diagnosis, pulmonary function tests (PFT) revealed a mixed restrictive-obstructive pattern, characterized by a reduced forced vital capacity (FVC 41% predicted) indicating restriction, together with reduced peak expiratory flow (PEF 67% predicted) and diminished mid-expiratory flows (MEF50 54%) consistent with an obstructive component. Chest radiography suggested a possible infectious process, for which oral amoxicillin was initiated. Around the same time, the patient experienced a clinical flare characterized by elevated inflammatory markers, weakness as evidenced by a worsening CMAS (40/52), and new symptoms of hoarseness and gastroesophageal reflux. At the time of this acute presentation of respiratory distress, he was still receiving prednisone 0.5 mg/kg/day. He received a repeated course of IV methylprednisolone (10 mg/kg/day for 3 days) and was maintained on high-dose oral prednisone (2 mg/kg/day) throughout the subsequent hospitalization. A repeat chest X-ray revealed new bilateral infiltrates, and a respiratory pathogen panel identified *Haemophilus influenzae*, prompting treatment with amoxicillin-clavulanic acid.

**Fig. 2:**
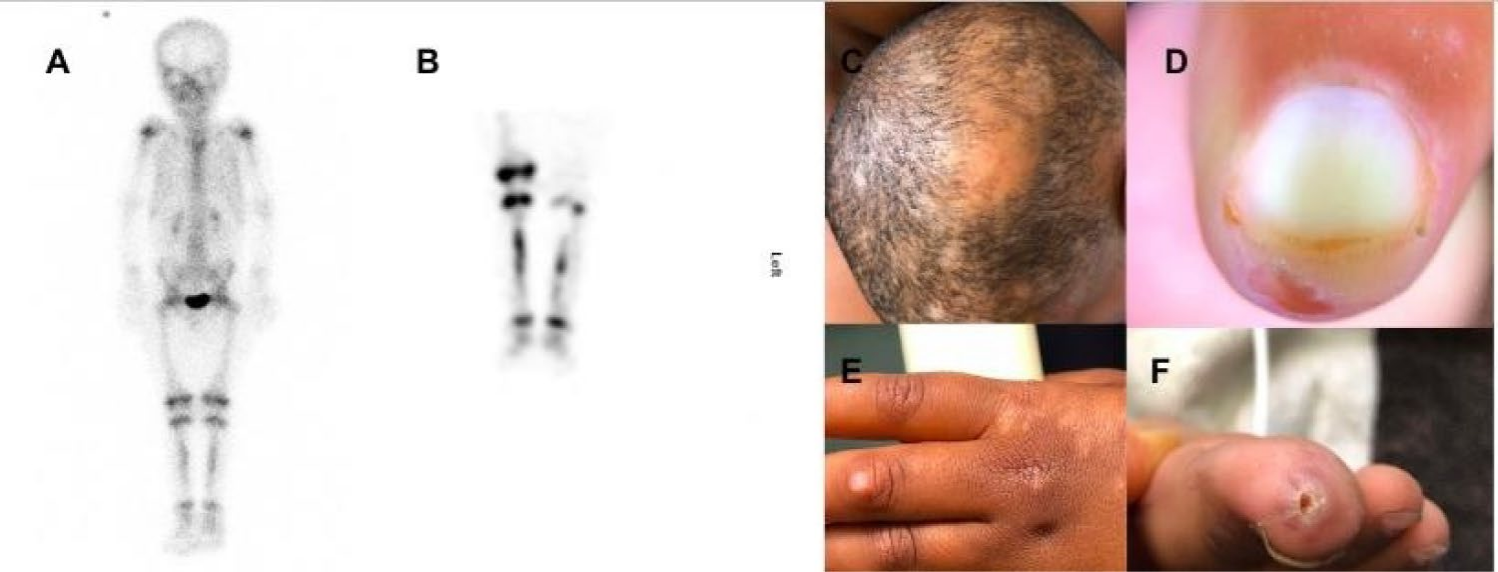
Baseline clinical and imaging findings in patient 2. **A** Whole-body scintigraphy showing increased radiotracer uptake in the upper limb musculature, chest wall, abdominal wall, elbow and wrist joints, and thighs. **B** SPECT imaging further confirming focal muscle inflammation with increased uptake. **C** Patchy alopecia. **D** Vasculitic lesion on the distal phalanx of the finger. **E** Gottron’s papule on the interphalangeal joint along with hypochromic lesions. **F** Vasculitic ulcerative lesion

One week after completing antibiotic therapy, the patient presented to the emergency department with acute respiratory distress and progressive swelling of the face and neck. Imaging revealed extensive pneumomediastinum, while laboratory workup showed elevated transaminases (ALT/AST), lymphopenia, and elevated creatine kinase (CK). Blood cultures and other septic markers were negative. A new infectious workup was conducted at this stage, which was negative, excluding other bacterial, viral, and fungal pathogens. Bronchoalveolar lavage was not performed due to the patient’s respiratory instability. Chest high-resolution computed tomography (HRCT) scan confirmed active, extensive interstitial lung disease (ILD) (Fig. 2). On day 2, cyclophosphamide (750 mg/m^2^) was initiated, and MMF was discontinued due to profound lymphopenia while corticosteroids were maintained at 2 mg/kg/day of IV methylprednisolone to ensure maximal anti-inflammatory effect (Table 1).

**Table 1:** Comparative laboratory and immunological profiles at diagnosis

Parameter	Patient 1	Patient 2	Patient 3	Reference range
Blood count and inflammatory markers				
Lymphocytes (× 10^9/L)	0.79	0.4	2.5	1.5–6.5
ESR (mm/h)	4	74	40	0–20
CRP (mg/L)	2	2	1	< 5
Ferritin (ng/mL)	463	64	919	7–140
Biochemistry				
CK (U/L)	Normal	Normal	Normal	20–200
LDH (U/L)	422	639	933	150–300
ALT (U/L)	69	76	124	10–50
AST (U/L)	70	111	317	10–55
Immunology				
Anti-MDA5	Positive	Positive	Positive	Negative
ANA	N/A	Negative	N/A	Negative

By day 4, the patient experienced rapid respiratory decline with tachypnea, severe asthenia, and difficulty speaking in full sentences. He was transitioned to high-flow oxygen via nasal cannula and admitted to the pediatric intensive care unit (PICU). Given the fulminant progression of respiratory involvement and the high mortality risk associated with rapidly progressive ILD in anti-MDA5 JDM, early therapeutic intensification was undertaken. Rituximab (375 mg/m^2^) was introduced in accordance with previously reported successful outcomes in refractory or severe cases [6].

Despite this escalation, the patient continued to deteriorate, requiring increased oxygen support. Ferritin levels were markedly elevated by day 12. Therapeutic plasma exchange, targeting 1 to 1.5 times the patient’s expected plasma volume per exchange, was initiated for five consecutive days. Due to the severity of lung disease and rapid deterioration, and considering case reports of efficacy, it was elected to add tofacitinib (5 mg twice daily). Following these interventions, the patient’s clinical condition gradually improved. Oxygen requirements decreased, ferritin levels normalized, and the neutrophil-to-lymphocyte ratio (NLR) approached baseline. He was discharged from the PICU on day 27 and transferred to the general ward, where he was progressively weaned to low-flow nasal cannula. On day 45, he was discharged home on oxygen therapy. Tacrolimus was initiated as a steroid-sparing agent in combination with tofacitinib.

At the 6-month follow-up, the patient’s respiratory status had improved, requiring nocturnal oxygen supplementation only. PFT revealed a persistent restrictive ventilatory defect, with a FVC reduced to 40% of the predicted value and a transfer coefficient for carbon monoxide (KCO) decreased to 44% of predicted. Steroid tapering was well tolerated from a respiratory standpoint, with a dose of 0.5 mg/kg/day reached at this time. Laboratory parameters showed normalization of ferritin levels and lymphocyte counts. However, from a muscular perspective, the patient remained steroid-dependent. Consequently, tacrolimus was discontinued, and immunosuppressive therapy was escalated to include monthly intravenous cyclophosphamide (750 mg/m^2^) and intravenous immunoglobulin (2 g/kg/month).

At the 6-month follow-up, the patient’s respiratory status had improved, requiring nocturnal oxygen supplementation only. PFT revealed a persistent restrictive ventilatory defect, with a FVC reduced to 40% of the predicted value and a transfer coefficient for carbon monoxide (KCO) decreased to 44% of predicted. Steroid tapering was well tolerated from a respiratory standpoint, with a dose of 0.5 mg/kg/day reached at this time. Laboratory parameters showed normalization of ferritin levels and lymphocyte counts, but elevated CK (240 U/L).

Attempts to taper below 0.5 mg/kg/day resulted in objective evidence of ongoing disease activity, including the emergence of new skin lesions and a CMAS score of 38/52. These findings indicated that the persistent weakness was more likely secondary to active inflammatory disease rather than steroid-induced myopathy. Consequently, tacrolimus was discontinued, and immunosuppressive therapy was escalated to include monthly intravenous cyclophosphamide (750 mg/m^2^) and intravenous immunoglobulin (2 g/kg/month).

#### Case 2

A 2-year 8-month-old boy with no significant past medical history presented to the emergency department with lower limb pain and failure to thrive. According to his grandmother, he had experienced frequent falls over the preceding 2 months, despite having previously attained independent ambulation. Additionally, there was concern about stagnation in weight gain.

On physical examination, the patient exhibited proximal muscle weakness, rendering him unable to stand without support. Given his age, a partial CMAS was performed, yielding a score of 30 out of 36. Musculoskeletal examination revealed arthritis affecting both knees and ankles. Dermatologic findings included vasculitic lesions on the digits, reverse Gottron’s papules, patchy alopecia, and hypopigmented macules in the axillary region (Fig. 2).

**Fig. 3:**
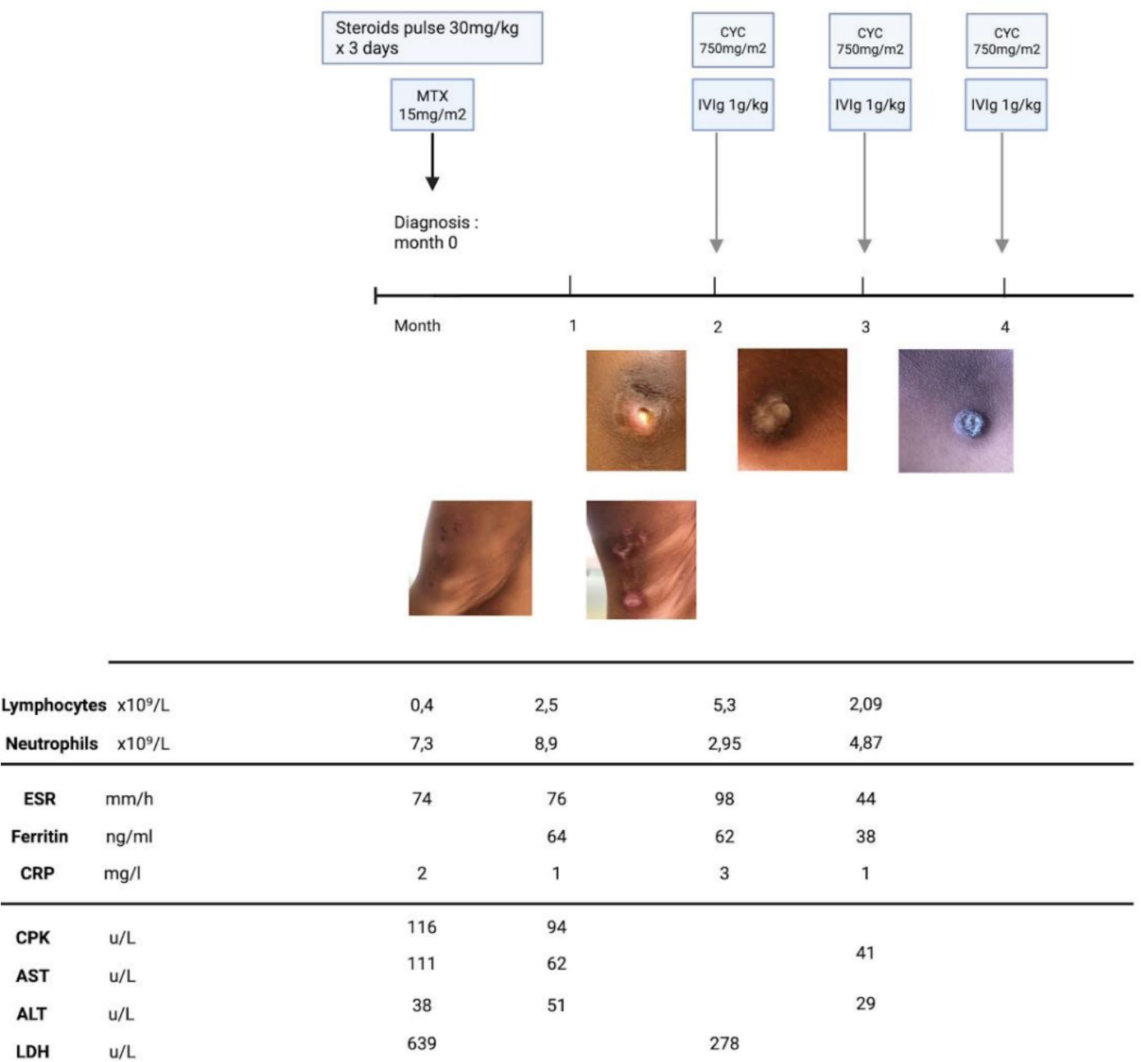
Clinical and biological evolution of patient 2

Initial laboratory investigations revealed an elevated erythrocyte sedimentation rate (ESR) with a normal C-reactive protein (CRP), marked lymphopenia, elevated ALT, normal CK, and elevated lactate dehydrogenase (LDH) (Fig. 3). Antinuclear antibodies (ANA) were negative. Extensive infectious workup was performed, and infections were ruled out, including tuberculosis, based on negative microbiological and radiologic findings. Nutritional deficiencies, particularly scurvy, were also considered but excluded (Table 1).

Myositis-specific autoantibody testing was positive for anti-MDA5 antibodies (immunoblotting). Due to the patient’s age, awake MRI was not feasible, and a bone scintigraphy was performed instead, revealing increased tracer uptake in multiple soft tissue regions, including the upper limb musculature, chest wall, upper back, abdominal wall, and thighs, consistent with inflammatory myopathy (Fig. 2). Consequently, a diagnosis of anti-MDA5-positive JDM was established.

The treatment was started with intravenous methylprednisolone (30 mg/kg/day for 3 days) followed by subcutaneous methotrexate (15 mg/m^2^ weekly).

**Fig. 4:**
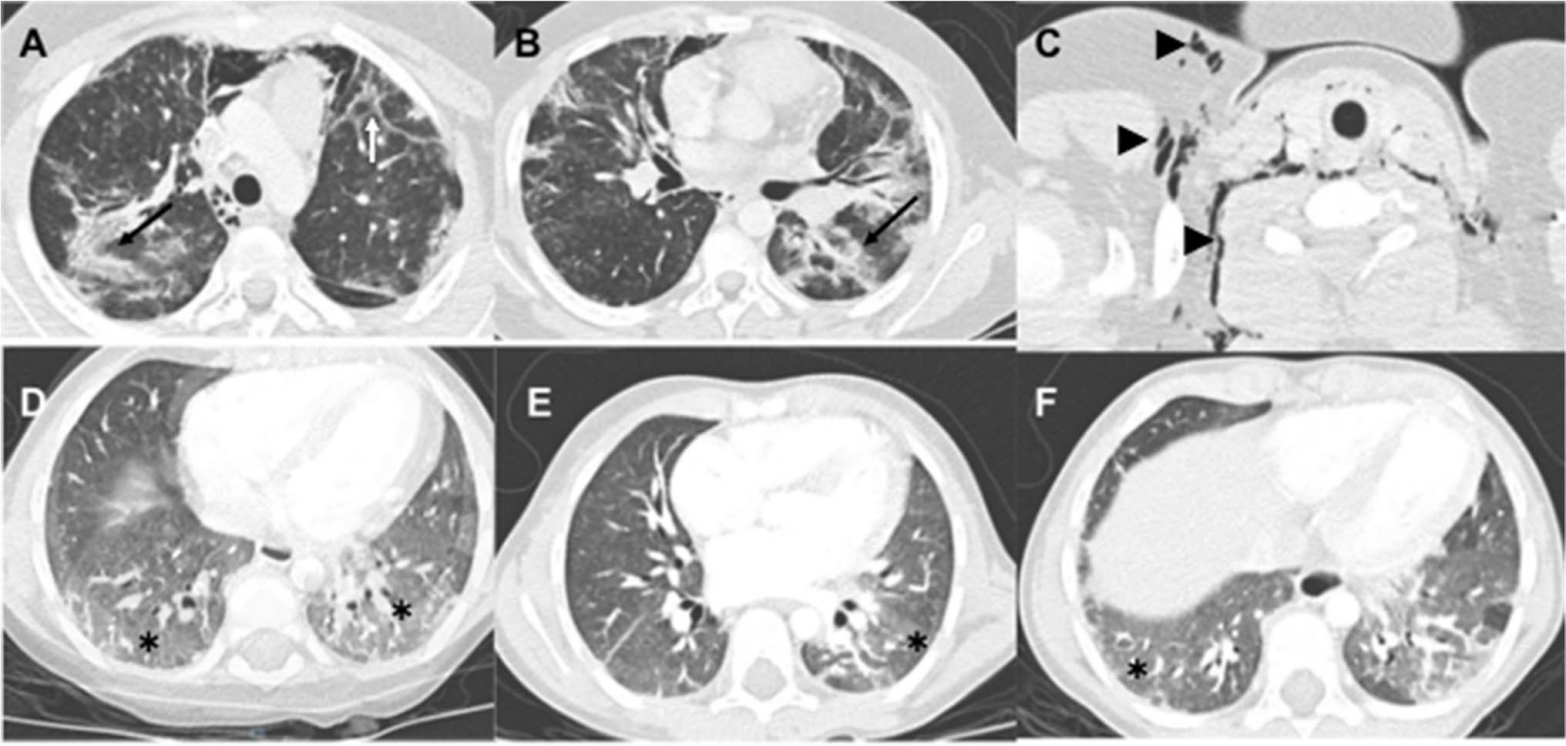
High-resolution computed tomography (HRCT) findings in two pediatric patients with anti-MDA5-positive juvenile dermatomyositis and interstitial lung disease. Patient 1: **A**–**B** Axial HRCT images from patient 1 demonstrates alveolar ground glass consolidation (black arrow) with smooth interlobular septal thickening (white arrow). **C** Axial HRCT demonstrates a pneumomediastinum (arrowhead) in the absence of pneumothorax, a known complication in severe ILD associated with anti-MDA5 autoantibodies. Patient 2: **D**–**F** Axial HRCT images from patient 2 demonstrates bilateral diffuse ground-glass opacification (asterisk)with areas of relative subpleural sparing.

After 1 month of treatment, the patient showed significant improvement in muscle strength and resolution of joint inflammation. However, the initially hypopigmented lesions in the axillary regions, previously managed with topical antifungal therapy, evolved into ulcerative lesions. Additionally, new ulcerative skin lesions developed over the knees and elbows. Although he remained asymptomatic from a respiratory standpoint, HRCT scan was performed due to the known association of anti-MDA5 antibodies with ILD. Imaging revealed subtle, diffuse ground-glass opacities with subpleural sparing, consistent with early pulmonary involvement in JDM (Fig. 4).

Given the severity of the cutaneous lesions and the presence of subclinical ILD, immunosuppressive therapy was escalated with the addition of intravenous cyclophosphamide (500 mg/m^2^) and monthly intravenous immunoglobulin (IVIG) at a dose of 2 g/kg, planned for 6 months. One month following this therapeutic intensification, no new skin ulcerations were observed, and existing lesions remained stable. By the second month, progressive healing of skin lesions was noted, and the patient remained free of respiratory symptoms with continued recovery of muscle strength (CMAS 36/36). Follow-up laboratory tests showed normalization of lymphocyte counts, improved liver function tests, and a near normalization of LDH levels.

#### Case 3

A 2-year 11-month-old girl presented to the emergency department with respiratory distress, tachycardia, and poor peripheral perfusion, with a background of being treated for respiratory infections in the months preceding presentation. She lived with her grandmother in a rural area, and her mother brought her to Cape Town for further evaluation after the child had received several courses of antibiotics with little improvement. There were no known tuberculosis contacts. She was fully immunized and had been growing appropriately for her age.

**Fig. 5:**
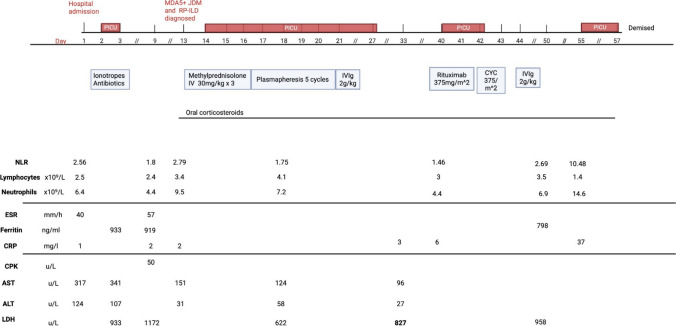
Clinical course of patient 3

She was admitted to ICU with a differential diagnosis of pneumonia, sepsis, and myocarditis. An urgent echocardiogram demonstrated a collapsed inferior vena cava, structurally normal intracardiac anatomy, and reduced myocardial contractility but a normal left ventricular ejection fraction of 56%. She was cautiously resuscitated with a fluid and started on peripheral ionotropic support, which was weaned within a short period, and she was transferred to the general pediatric ward on high-flow oxygen therapy. Viral respiratory studies were positive for respiratory syncytial virus. A repeat echocardiogram showed normal cardiac function (Fig. 5).

Despite completing an adequate course of antibiotics, she failed to improve clinically, with increasing oxygen requirements and progressive changes indicative of interstitial lung disease on chest radiography. Throughout this period, septic markers remained low, with consistently normal C-reactive protein. An extensive tuberculosis work-up was negative at that time.

A more detailed history obtained from her grandmother revealed that over the preceding year, the child had experienced intermittent facial swelling and episodic facial rash, along with increasing difficulty rising from the floor, difficulty running, and frequent falls. There were also episodes of painful swelling of the knees and ankles, which resolved spontaneously. On careful examination, a subtle violaceous peri-orbital rash consistent with a heliotrope-type rash was noted, in addition to subtle vasculitis over her fingertips with scars from possible ulcerative lesions over extensor surfaces. She had a generalized hyperpigmented macular rash and excoriated lesions over her elbows. Musculoskeletal examination revealed bilateral ankle swelling and pain on flexion of the knees, wrists, and ankles. Proximal muscle weakness was evident, and she struggled to sit up from a supine position. Nailfold capillaroscopy showed dilated capillary loops. Blood investigations showed a raised ferritin with persistently elevated LDH. The myositis antibody panel was strongly positive for MDA-5 antibodies (immunoblot), and muscle MRI confirmed inflammatory myositis with extensive subcutaneous inflammation (Table 1).

**Fig. 6:**
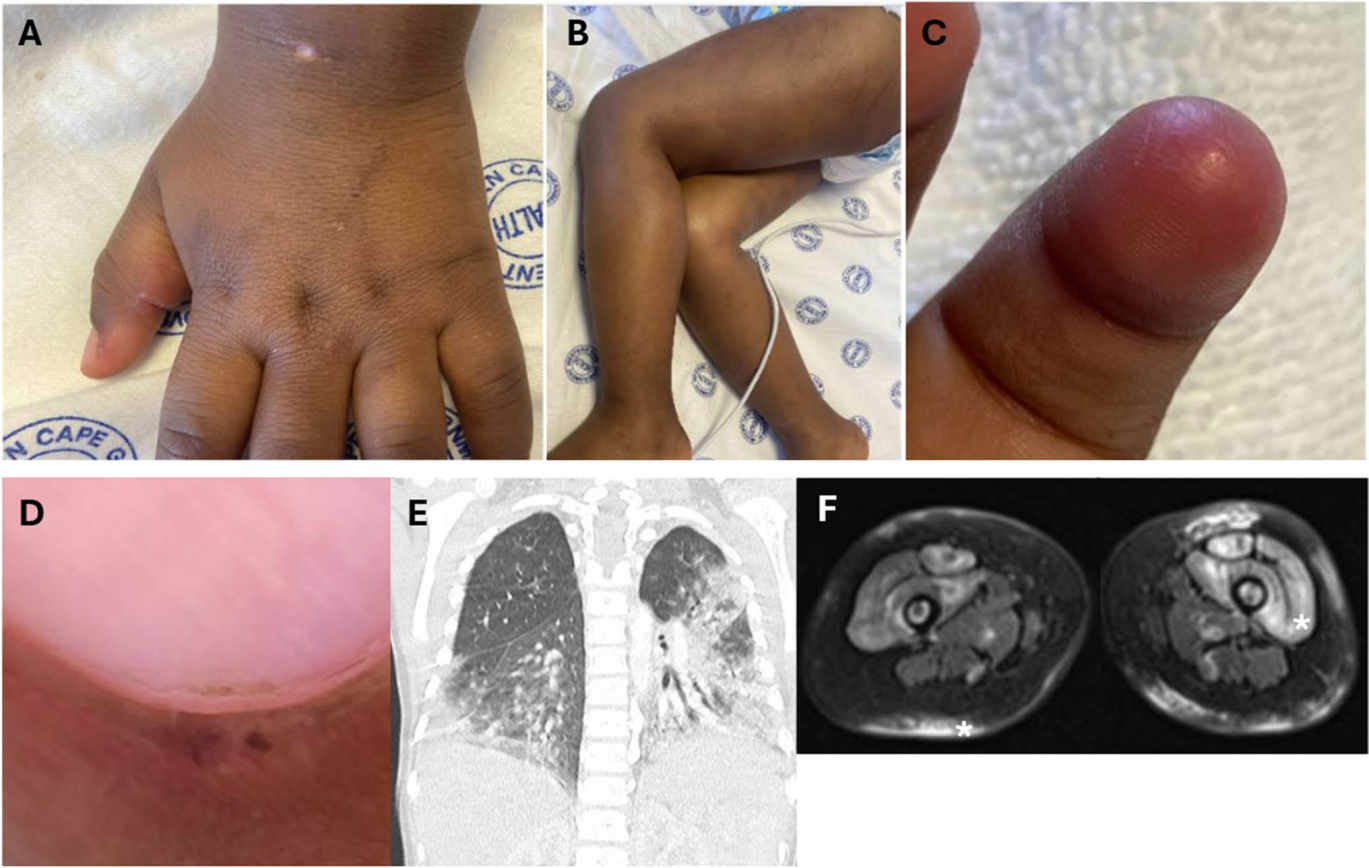
Clinical and imaging findings in patient 3. **A** Ulcer over the dorsal aspect of the wrist. **B** Hyperpigmented macular rash with mild excoriation. **C** Vasculitic rash over fingertips. **D** Subtle capillary loop dilation and small infarct visible on nail bed capillaroscopy. **E** Coronal CT chest demonstrates bilateral lower lobe and lingula confluent ground glass opacification with left lower lobe traction bronchiectasis. **F** MRI showing T2 axial and coronal fat sat images that demonstrate extensive high T2 hyperintense signal abnormality in the muscles and subcutaneous tissues of the thighs bilaterally (white asterisks)

A diagnosis of MDA5 + JDM with RP-ILD was made, and she was readmitted to PICU. She received a 3-day pulse of intravenous methylprednisolone (30 mg/kg/day), followed by five cycles of plasmapheresis and subsequent intravenous immunoglobulin (2 g/kg). In PICU, she developed a central line-associated sepsis with *Candida* species and *Staphylococcus aureus* which responded to antibiotic therapy (Fig. 6).

After she had recovered from sepsis, she received one dose of rituximab (375 mg/m^2^) and cyclophosphamide (375 mg/m^2^). Her clinical condition continued to deteriorate. Bronchoscopy with bronchoalveolar lavage demonstrated acid-fast bacilli, and cultures confirmed *Mycobacterium avium* complex, for which appropriate treatment was initiated. Two weeks later, she developed a spontaneous pneumomediastinum and severe respiratory deterioration, requiring PICU admission bilateral chest drain insertion and intubation with high-frequency oscillatory ventilation. Despite maximal medical and intensive care support, the child died 2 months after admission.

## Literature review

A comprehensive literature search was conducted using the PubMed database for articles published between January 1, 1997, and March 31, 2025, employing the search terms:

“Juvenile dermatomyositis” *AND *“rapidly progressive interstitial lung disease” *AND *(“MDA5” *OR *“anti-MDA5” *OR *“CADM-140”).

The primary criteria for inclusion in this case-based review were as follows:

- Confirmed pediatric cases (age < 18 years) meeting the Bohan and Peter or EULAR/ACR criteria for JDM.
- Positive testing for anti-MDA5 (formerly anti-CADM-140) antibodies.
- Documentation of RP-ILD, defined as a progressive worsening of radiological interstitial changes and/or dyspnea within 3 months of the onset of respiratory symptoms.
- Availability of detailed therapeutic interventions and clinical outcomes.

Due to the absence of therapeutic details, one article describing three fatal cases of RP-ILD in young girls (aged 4, 4, and 6) was omitted from the summary table [7]. Ultimately, 11 articles reporting 17 cases were selected.

Analysis of the published cases revealed considerable heterogeneity in clinical presentation, treatment approaches, and outcomes. Most patients were female, with a median age at diagnosis of approximately 8 years. All reported cases featured classic cutaneous manifestations—such as Gottron’s papules, ulcerations, or malar rash—commonly accompanied by proximal muscle weakness and weight loss. Pulmonary involvement typically developed within the first 3 months of symptom onset, often in the absence of overt respiratory symptoms.

Laboratory findings frequently included markedly elevated levels of ferritin, lactate dehydrogenase (LDH), and KL-6, supporting their use as biomarkers for disease activity and prognosis. The co-expression of anti-Ro52 antibodies was noted in some patients and appeared to be associated with a more severe disease course.

Therapeutic strategies varied among cases but consistently involved early, intensive immunosuppressive regimens initiated at the time of diagnosis. Eight of the 17 reported cases resulted in early fatalities, highlighting the potentially fulminant nature of RP-ILD in anti-MDA5-positive JDM.

This review underscores the importance of early recognition and aggressive, multimodal therapy to improve survival in this rare but life-threatening subset of juvenile dermatomyositis. Detailed findings from these reports are summarized in Table 2.

**Table 2:** Published case reports of anti-MDA5 positive juvenile dermatomyositis with rapidly progressive interstitial lung disease: a comparative summary

Author	Year	Country	N	Sex	Age at Dx	ILD at Dx	Age at RP	MuscleW	skin Ulcers	Arthritis
Kobayashi et al [8]	2011	Japan	3	2 M/F	7–14	Yes	+1–2 mo	Yes	Yes	No
Sakurai et al [9]	2011	Japan	1	1 M	9	Yes	+6 mo	Yes	No	No
Hou et al [10]	2019	China	1	1 F	10	Yes	NA	Yes	No	No
Quintana et al [11]	2020	Spain	1	1 F	11	Yes	+5 mo	Yes	No	Yes
Shimizu et al [12]	2021	Japan	1	1 F	2	Yes	+2 mo	Yes	No	No
Yeung et al [13]	2021	China	1	1 F	16	Yes	+2 mo	Yes	No	No
Nishi et al [6]	2022	Japan	1	1 F	2	Yes	+6 days	Yes	No	No
Ciaglia et al [14]	2024	USA	1	1 F	4	Yes	+3 mo	Yes	Yes	Yes
Qui et al [15]	2024	China	1	1 F	3	Yes	+3 mo	Yes	Yes	No

Author	Pneumomediastinum	Ferritin	LDH	KL6	CK	Ro52	RP-ILD Treatment	Intubation	ECMO	Outcome
Kobayashi et al [8]	No	NA	NA	2460-2376	1250-3484	No	CS+MMF/CSA	No	No	1 death/ 2 remission
Sakurai et al [9]	No	2006	407	NA	858	No	CS+CYC+IVIG+RTX	Yes	Yes	Alive O2 dependent
Hou et al [10]	No	NA	576	3420	N	No	CS+CSA+CYC+IVIG+PE+RTX	Yes	No	Alive
Quintana et al [11]	No	447	749	NA	N	Yes	CS+JAKi+IVIG+RTX	No	No	Alive
Shimizu et al [12]	No	300	736	NA	485	No	CS+RTX+PE	Yes	No	Died
Yeung et al [13]	Yes	5134	NA	NA	NA	No	CS+CYC+ JAKi +PE	Yes	No	Died
Nishi et al [6]	No	130	564	5604	48	No	CS+RTX	No	No	Alive
Ciaglia et al [14]	No	NA	480	2703	NA	No	CS+CYC+TAC+PE	NA	NA	Alive
Qui et al [15]	No	1792	NA	NA	588	Yes	CS+JAKi	NA	NA	Died

*ALT* alanine aminotransferase (N 10–50 U/L), *AST* aspartate aminotransferase (N 10–55 U/L), *CK* creatine kinase (N 20–200 U/L), *CS* corticosteroids, *CSA* cyclosporine, *CYC* cyclophosphamide, *Dx* diagnosis, *EF* ejection fraction, *ESR* erythrocyte sedimentation rate (N 0–20 mm/h), *F* female, *Ferr* ferritin (N 7–140 pmol/L), *HCQ* hydroxychloroquine, *ILD* interstitial lung disease, *IVIG* intravenous immunoglobulin, *JDM* juvenile dermatomyositis, *KL6* Krebs von den Lungen-6 (lung injury marker, N < 500 U/mL), *LDH* lactate dehydrogenase (N 150–300 U/L), *MDA5* melanoma differentiation-associated gene 5 antibody, *MMF* mycophenolate mofetil, *MTX* methotrexate, *N* normal, *PE* plasma exchange, *Ro52* antibody, *RP-ILD* rapidly progressive interstitial lung disease, *RTX* rituximab, *TAC* tacrolimus, *TAG* triglycerides (*N* < 150 mg/dL), *W* weakness

## Discussion

JDM associated with anti-MDA5 autoantibodies is a rare and clinically heterogeneous subtype of inflammatory myopathies. The clinical course of anti-MDA5 JDM remains unpredictable, and while the literature is predominantly based on adult cohorts, it consistently reports high early mortality rates, frequently within the first 6 months post-diagnosis, primarily attributable to RP-ILD [16]. Our series of the first three documented African cases highlights several key issues. First, the distinctive clinical phenotype in children. Second, it raises the question of environmental triggers, including SARS-CoV-2. Third, it underscores the need for early aggressive “triple therapy”. Finally, it reinforces the necessity of screening for subclinical lung involvement.

A nuanced understanding of anti-MDA5-associated disease necessitates distinguishing its adult and pediatric manifestations. Muscle involvement represents a key point of divergence: adults most often present with an amyopathic or hypomyopathic phenotype, whereas children more frequently exhibit clear muscle weakness. Nevertheless, amyopathic or hypomyopathic dermatomyositis can occur in pediatric patients and may lead to diagnostic delays, which may bring complications that are not frequently observed in these patients, including macrophage activation syndrome (MAS) [17, 18]. MAS is clinically characterized by sustained fever, hepatosplenomegaly with laboratory features including cytopenias, extreme hyperferritinemia, elevated liver enzymes. In the context of juvenile dermatomyositis (JDM), MAS often emerges at disease onset frequently before a definitive diagnosis is established and may be under-recognized due to overlapping clinical features. Its potential to occur throughout the disease course makes vigilant monitoring essential [19].

Arthritis also differs across populations, occurring more commonly in pediatric patients, while being less frequently observed in adults. The most clinically significant distinction, however, lies in the pattern and severity of ILD. Adults carry a substantially higher risk of RP-ILD, which accounts for the high early mortality seen in this population. Although children similarly demonstrate an elevated overall risk of ILD, RP-ILD appears to be less common in pediatric cohorts. Nonetheless, when RP-ILD does occur as illustrated by our first and third cases, the clinical course can be equally severe. This underscores the particular challenge of pediatric anti-MDA5 JDM: despite a lower overall likelihood of RP-ILD compared with adults, its occurrence demands prompt and intensive management to avert life-threatening progression [4, 20].

Second, environmental and host-related factors may contribute to disease onset and progression. A North American cohort has suggested associations with recent stressful life events, ultraviolet radiation exposure, lower population density, and proximity to water sources [4]. In this case, the occurrence of three anti-MDA5 JDM cases within such a short period is consistent with recent evidence showing that SARS-CoV-2 infection can induce anti-MDA5 autoantibody production and increase the incidence of MDA5-associated disease. This aligns with reports that COVID-19 driven type-I interferon overactivation may unmask or accelerate MDA5-mediated autoimmunity in genetically predisposed individuals [21]. Elements warrant further exploration in future studies.

Third, early recognition of high-risk patients is crucial to guide prompt therapeutic escalation. Elevated anti-MDA5 antibody titers and high serum ferritin levels have been consistently linked to an increased risk of rapidly progressive ILD and poor outcomes [16]. KL-6 has also emerged as a potential biomarker for ILD severity and progression, aligning with observations in adult ILD, where both anti-MDA5 titers and KL-6 levels have been associated with relapse risk [7, 22]. Co-positivity for Ro52 antibodies may further contribute to disease severity [23].

At present, management of pediatric anti-MDA5 JDM relies heavily on adult-derived data and expert consensus. The most widely adopted regimen referred to as “triple therapy,” comprising high-dose glucocorticoids, a calcineurin inhibitor, and intravenous cyclophosphamide, has demonstrated improved outcomes in adult populations [24]. More recently, Janus kinase (JAK) inhibitors have shown promise due to their targeted inhibition of type-I interferon signaling, a pathway implicated in the pathogenesis of anti-MDA5 JDM [25].

In our first patient with RP-ILD, early initiation of an intensive immunosuppressive regimen including high-dose corticosteroids, cyclophosphamide, rituximab, plasmapheresis, and JAK inhibition was instrumental in controlling the pulmonary manifestations. Despite an initial deterioration requiring PICU admission and the initiation of plasmapheresis, the patient ultimately stabilized. Ferritin and the neutrophil-to-lymphocyte ratio (NLR) were used to monitor disease activity and therapeutic response, with their gradual normalization paralleling the clinical improvement.

To our knowledge, we report here the 18th and 19th cases of MDA5 + JDM with RP-ILD globally. These three cases represent the first reported instances of anti-MDA5-positive JDM on the African continent. This is of particular importance given that the current literature is largely limited to cohorts from East Asia, Europe, and North America. Expanding the geographical representation of this disease helps broaden our understanding of its clinical spectrum and reinforces the need for diagnostic capacity and awareness in resource-limited settings.

These cases illustrate the marked variability in clinical presentation and disease trajectory. While two patients developed RP-ILD necessitating aggressive immunosuppressive therapy and intensive care support and varying outcomes, the other presented with prominent cutaneous manifestations and a more indolent respiratory course. Skin ulcerations associated with anti-MDA5 usually manifest as deep, painful ulcers on the joint and on the digital pulp. Histologically, these lesions show features of vasculopathy. Less specific auricular skin lesions have also been described [1, 26]. In our second patient, ulcerative lesions were typical of anti-MDA5 JDM, with necrotic ulcers overlying extensor surfaces and erythematous papules on the antihelix, associated with patchy alopecia, findings consistent with the vasculopathic and heterogeneous cutaneous manifestations characteristic of this subtype.

It is noteworthy that in case 2, lung involvement was asymptomatic at presentation. This highlights the potential for subclinical pulmonary disease and underscores the importance of routine screening with HRCT and PFT, even in the absence of respiratory symptoms. Such variability reinforces the need for early recognition, close monitoring, and individualized management strategies.

In conclusion, these cases highlight the critical need for standardized treatment algorithms and the development of international collaborative registries dedicated to pediatric anti- MDA5 JDM. Early identification of high-risk patients using clinical, serologic, and radiologic markers remains essential to improving outcomes. Prospective studies are urgently needed to establish evidence-based treatment protocols for this vulnerable population.

## Data Availability

Not applicable.
